# Bactericidal Activity of Pradofloxacin and Other Antimicrobials Against Swine Respiratory Bacterial Pathogens

**DOI:** 10.3390/pathogens14111171

**Published:** 2025-11-17

**Authors:** Joseph M. Blondeau, Shantelle D. Fitch

**Affiliations:** 1 Department of Clinical Microbiology, Royal University Hospital and Saskatchewan Health Authority, Saskatoon, SK S7N 0W8, Canada; shantelle.fitch@saskhealthauthority.ca; 2 Departments of Biochemistry, Microbiology and Immunology, Pathology and Laboratory Medicine and Ophthalmology, University of Saskatchewan, Saskatoon, SK S7N 0W8, Canada

**Keywords:** swine, bacterial infection, pradofloxacin

## Abstract

Swine respiratory disease (SRD) is a complex interaction whereby viral infection predisposes the host to secondary bacterial pulmonary invasion, which may be fatal. Antimicrobial agents remain an important therapy and serve to reduce morbidity and mortality in treated animals. Pradofloxacin is the newest of the veterinary antibiotics to be approved to treat SRD. It is a dual-targeting fluoroquinolone with in vitro and clinical activity against Gram-negative and -positive bacteria, along with atypical agents including anaerobes. In this study, we compared the killing of *Actinobacillus pleuropneumoniae*, *Pasteurella multocida,* and *Streptococcus suis* by pradofloxacin and comparator antibiotics in a 3 h kill assay, using four clinically relevant drug concentrations. Pradofloxacin was bactericidal against the three pathogens, with kill rates ranging from 94.4 to 99.9% (*A. pleuropneumoniae*) following 15–20 min of exposure to the maximum serum and maximum tissue drug concentration. For *P. multocida*, the kill rates were 68.7–96.9% following 5–30 min of drug exposure at the maximum serum drug concentration, and 91.7% following 5 min of drug exposure at the maximum tissue drug concentration. For *S. suis*, pradofloxacin killed 92.4–99.4% and 71.6–97.1% of cells following 60–180 min of drug exposure at the maximum serum and maximum tissue drug concentration, respectively. Pradofloxacin appears to be an important addition to the drugs currently available for treating SRD.

## 1. Introduction

Swine respiratory disease is an expensive infectious complication of pig farming, costing in excess of a billion dollars annually [1]. Porcine respiratory disease complex defines the complex interactions between viruses such as Porcine Reproductive and Respiratory Syndrome virus (PRRSV), Swine Influenza virus (SIV), Porcine Respiratory Coronavirus (PRCV), Pseudorabies virus (PRV), and Porcine Circovirus (PcV_2_), opportunistic bacterial pathogens including *Mycoplasma hyopneumoniae*, *Bordetella bronchiseptica*, *Pasteurella multocida*, *Actinobacillus pleuropneumoniae*, *Glaesserella parasuis*, and *Streptococcus suis*, and management and environmental conditions including overcrowding, ventilation, temperature, mixing of animals from different sources, continuous flow, and sanitation. Considering the pathogenesis, viruses and primary bacterial pathogens infecting pigs serve to weaken immune defences and predispose animals to opportunistic secondary bacterial infections [2]. *A. pleuropneumoniae* is considered a primary bacterial pathogen of swine, is found globally, and is associated with fatal infections [3]. *P. multocida* has been recognized as a contributor of debilitating and fatal pig pneumonia for ~120 years and may be related, in part, to virulence factors including biofilm formation [4]. *S. suis* is associated with respiratory disease, meningitis, arthritis, and sudden death, and is often considered a co-pathogen in mixed infections [5]. Dosen et al. reported on bacterial infection of the respiratory tract of swine [6]. Of 125 pigs that had died of presumed respiratory tract infection and from which lungs and mediastinal lymph nodes were examined, *Pasteurella* spp. was the most frequently isolated at 26.57%, compared to 3.5% for *A. pleuropneumoniae* and 1.4% for *S. suis*.

Sargent, in a systemic review of antibiotics for the prevention of swine respiratory disease, indicated circumstances under which antibiotics are used: (1) individual animals showing clinical signs of infection, (2) prophylactic treatment of groups of animals to prevent infection and clinical disease where there is a known risk and (3) metaphylactically in groups of animals where some animals may already be infected and to prevent further spread within the group [7]. Vaccine strategies have shown encouraging results against some pathogens associated with swine respiratory disease complex, but these vaccines do not cover all bacterial pathogens, thereby continuing the need for antibiotics as therapy interventions [8,9]. Understanding the in vitro characteristics of antibiotics is important for correlating in vivo observations from clinical studies with routine clinical use.

In vitro kill measurements can differentiate antibiotics into categories such as bactericidal or bacteriostatic [10] and determine the rate and extent of killing under controlled conditions [11]. Additionally, the impact of drug concentrations and duration of drug exposure can be investigated. To the best of our knowledge, a head-to-head comparison of in vitro killing of swine bacterial pathogens by approved antimicrobial agents has not previously been performed. The purpose of this study was to determine the speed and extent of in vitro killing by pradofloxacin against swine bacterial pathogens and key results compared to those of ceftiofur, danofloxacin, enrofloxacin and marbofloxacin against swine isolates of *A. pleuropneumoniae* and *S. suis*; pradofloxacin to ceftiofur, enrofloxacin, florfenicol, marbofloxacin, tildipirosin, tilmicosin and tulathromycin against swine isolates of *P. multocida* in a 3 h kill assay. Pradofloxacin was rapidly bactericidal against the various pathogens tested; the rapid bactericidal activity of pradofloxacin may be clinically useful, and its dual-targeting mechanism of action may prevent resistance selection.

## 2. Materials and Methods

### 2.1. Bacterial Strains

Matrix-assisted laser desorption ionization—time of flight (MALDI-TOF) (BioMerieux, St. Laurent, QC, Canada) and/or Vitek II (BioMerieux, St. Laurent, QC, Canada) were used to confirm random genetically uncharacterized strains of *A. pleuropneumoniae* (n = 3), *P. multocida* (n = 3), and *S. suis* (n = 3), generously provided by Purdue University (West Lafayette, IN, USA). Isolates were plated on tryptic soy agar containing 5% sheep red blood cells (BA) or chocolate agar plates (CA) (*A. pleuropneumoniae*) (Oxoid, Nepean, ON, Canada) in O_2_ (CO_2_ for *A. pleuropneumoniae* and *S. suis*) at 35–37 °C for 18–24 h. Following incubation, single colonies were selected and transferred to skim milk using a sterile wooden stick and stored frozen at −70 °C. Each wild-type isolate had to be susceptible to each drug based on the currently available recommended susceptibility MIC breakpoints [12], but no pre-selection criteria favoured the inclusion of organisms with specific susceptibility (i.e., lower or higher MIC values) to any drug tested.

### 2.2. Antimicrobial Compounds

Pure enrofloxacin and pradofloxacin were obtained from Elanco (Greenfield, IN, USA) and prepared as per the manufacturer’s instructions. Ceftiofur and danofloxacin (Zoetis, Kirkland, QC, Canada), florfenicol (Merck, Kirkland, QC, Canada), marbofloxacin (Veto quinol, Laval Trie, QC, Canada), tildipirosin (Merck Kirkland, QC, Canada), and tilmicosin and tulathromycin (Zoetis, Kirkland, QC, Canada) were purchased commercially and prepared in accordance with the manufacturer’s directions. Fresh stock solutions or samples stored at −70 °C were used for each experiment.

### 2.3. Minimum Inhibitory Concentration (MIC) Testing

MIC testing followed the recommended protocol by the Clinical and Laboratory Standards Institute [13], whereby thawed isolates were sub-cultured on BA (Thermo Fisher, Nepean, ON, Canada) (*P. multocida*, *S. suis*) or CA plates (Thermo Fisher, Nepean, ON, Canada) (*A. pleuropneumoniae*) for 18–24 h at 35–37 °C in O_2_ or CO_2_ (*A. pleuropneumoniae* and *S. suis*). All isolates were tested independently. Mueller-Hinton Broth (MHB) (*P. multocida*) (Thermo Fisher, Nepean, ON, Canada), MHB with yeast extract (*A. pleuropneumoniae*) (Thermo Fisher, Nepean, ON, Canada), or MHB with 3% laked horse blood (Thermo Fisher, Nepean, ON, Canada) (*S. suis*) containing 2-fold drug concentration increments was added to 96-well micro dilution trays. Drug concentration ranges from 0.001 to 128 µg/mL were used, and the range varied depending on the specific drug tested. *A. pleuropneumoniae*, *P. multocida,* and *S. suis* isolate suspensions equal to a 0.5 McFarland standard were diluted in the appropriate MHB to a final inoculum of 5 × 10^5^ cfu/mL and added to microtiter trays, incubated for 18–24 h at 35–37 °C (O_2_ or CO_2_ as appropriate), following which the lowest drug concentration preventing visible bacterial growth was recorded as the MIC. The American Type Culture Collection (ATCC) strains *Enterococcus faecalis* 29212, *Escherichia coli* 25922, *Staphylococcus aureus* 29213 and *Pseudomonas aeruginosa* 27853 were tested with each MIC assay to ensure the assays were within acceptable performance ranges.

### 2.4. Mutant Prevention Concentration (MPC) Testing

Five BA (*P. multocida*), 10 BA (*S. suis*), or 5 CA plates (*A. pleuropneumoniae*) per isolate were inoculated for confluent growth and incubated for 18–24 h at 35–37 °C in O_2_ or CO_2_ (as appropriate), following which the complete plate contents of bacterial growth were transferred to 100 mL of MHB and incubated for 18–24 h at 35–37 °C (O_2_ or CO_2_ as appropriate) [14,15]. *A. pleuropneumoniae* and *S. suis* cultures were centrifuged at 3300 rpms for 30 min at 4 °C. Cultures were estimated to have concentrations of ≥3 × 10^9^ cfu/mL by spectrophotometric readings (600 nm) ≥ 0.3 (Thermo Scientific Genesys 10s vis, Mississauga, ON, Canada) and by colony counts. Aliquots of 100 µL containing ≥10^9^ cfu were applied to antimicrobial-containing BA plates over a range of drug concentrations from one dilution below the measured MIC to the determined MPC value. Drug plates were used within 1 week of preparation. Inoculated plates were incubated as described, with examination for growth at 24 and 48 h. All strains were tested independently. The lowest drug concentration preventing growth (48 h) was the MPC. Each experiment included the 4 ATCC control strains summarized above. The high bacterial density used for MPC testing was not used in kill assays—only to determine MPC values.

### 2.5. Kill Experiments

*A. pleuropneumoniae* (4 CA plates), *P. multocida* (4 BA plates), and *S. suis* (1½ BA plates) isolates were inoculated and incubated for 18–24 h at 35–37 °C (O_2_ or CO_2_ as appropriate), following which an inoculum was transferred to the most appropriate MHB and incubated at 35–37 °C in O_2_ or CO_2_ for 2 h, and spectrophotometric readings of ≥0.3 verified cell densities ≥10^9^ cells/mL [14]. Subsequent adjusting of inoculate to achieve cell densities of 10^5^ cfu/mL was the most appropriate in MHB to which the antimicrobial agent was added.

Antimicrobial concentrations used in kill experiments were based on measured MIC or MPC drug concentrations for each antimicrobial agent against each strain. The maximum plasma (C_max_) and maximum tissue (T_issuemax_) drug concentrations were from published studies or reports for ceftiofur, enrofloxacin, florfenicol, marbofloxacin, pradofloxacin, tildipirosin, tilmicosin, and tulathromycin (Table 1) [16]. Killing (log_10_ reduction in viable cells and percentage of organism killed) was recorded at 5, 10, 15, 20, 25, 30, 60, 120, and 180 min following drug exposure by culturing aliquots on drug-free blood agar or chocolate agar plates incubated for 18–24 h at 35–37 °C in O_2_ or CO_2_. Bacterial killing was quantified by measuring the reduction in viable cell count from time 0 to the count at time 5 min after drug exposure, and so on, which were tested independently. Three separate aliquots were sampled at each time frame and the results were averaged, as were the results for the 3 strains of each genus so that each data point on the log_10_ reduction graphs represents the average of 9 individual measurements (i.e., measurements in triplicate and averaged for 3 strains).

### 2.6. Statistical Analysis

Statistical analysis of the data was performed by means of a repeated-measures ANCOVA for each drug/log-exposure dataset, with fixed effects consisting of drug, exposure time, and drug-by-time interaction. In each model, the CFU count at time 0 was included as a covariate, and a compound symmetric covariance structure was used. The CFU counts were logarithmically transformed to achieve a normal distribution. Bonferroni adjustments for multiple comparisons were made. Values of *p* ≤ 0.05 were considered significant for all analyses.

We investigated the dependence on time. For this, we fitted a repeated measures model with covariate (Time 0), treatment, time, and treatment-by-time interactions as fixed effects. The time factor in the model measures whether the mean response differs over time. Also, the treatment-by-time interaction term tests whether the groups’ mean responses change over time. Significant change over time for these factors is declared if the *p* value < 0.053.

## 3. Results

### 3.1. Actinobacillus pleuropneumoniae

**Figure 1:**
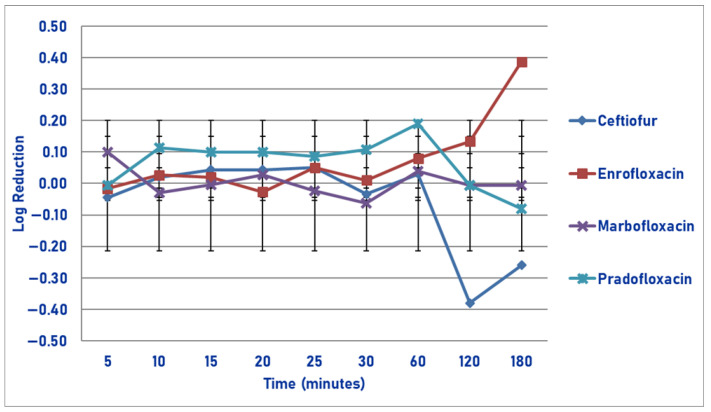
Log reduction in *Actinobacillus pleuropneumonia* (Isolates Averaged) by 4 antimicrobial agents—MIC.

At the drug MIC, killing was poor for all drugs tested and was highest for ceftiofur following 120 min of drug exposure (48.6% kill), following which regrowth occurred (Figure 1). No significant differences were seen in killing between any of the agents tested.

**Figure 2:**
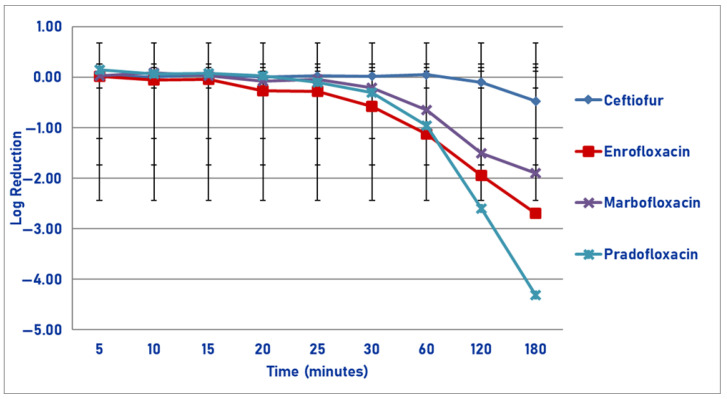
Log reduction in *Actinobacillus pleuropneumonia* (Isolates Averaged) by 4 antimicrobial agents—MPC.

At the drug MPC and following 60–120 min of drug exposure, pradofloxacin killed between 72.1 and 99.7% of cells compared to growth −3.5% for ceftiofur, 86.5–98.9% for enrofloxacin, and 26.8–82.3% for marbofloxacin (Figure 2). Following 180 min of drug exposure, pradofloxacin killed 97.9% of cells versus 54.8% for ceftiofur, 99.8% for enrofloxacin, and 91.4% for marbofloxacin. Statistically significant differences in killing between the agents tested were not seen at any time point measured. Statistically significant differences were seen for time (*p* < 0.0001) and treatment by time (*p* < 0.0001).

**Figure 3:**
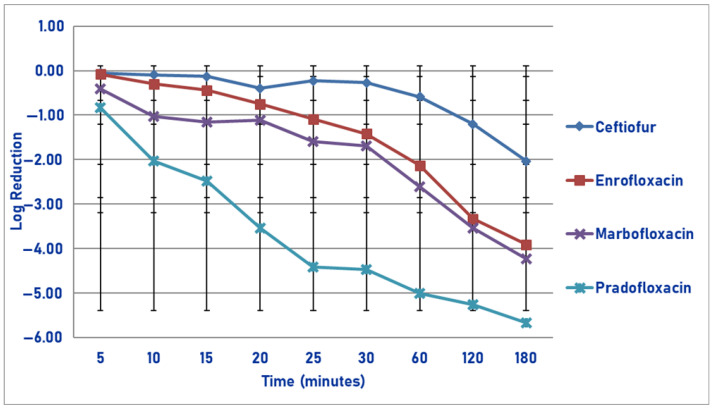
Log reduction in *Actinobacillus pleuropneumonia* (Isolates Averaged) by 4 antimicrobial agents—C_max_.

Following exposure to the maximum plasma drug concentration, pradofloxacin killed 79.0% of cells following 5 min of drug exposure, which increased to 99.9% following 20 min and 100% kill following 120 min (Figure 3). By comparison, at 5, 20, and 180 min after drug exposure, ceftiofur killed 10.9, 58.9, and 78.8% of cells; enrofloxacin killed 17.5, 79.5, and 99.9% of cells; and marbofloxacin killed 60.1, 86.8, and 99.9% of cells. Statistically significant differences in killing between the agents were not seen following 5 and 10 min of drug exposure. Following 15–180 min of drug exposure, pradofloxacin (2.5–5.7 log_10_, 99.4–100% kill) killed more cells than ceftiofur did (0.1–2.0 log_10_, 24.3–78.8% kill, *p* values 0.0169–<0.0001). Following 20–60 min of drug exposure, pradofloxacin (3.5–5.0 log_10_, 99.9–99.99% kill) killed more cells than enrofloxacin did (0.8–2.1 log_10_, 79.5–98.7% kill, *p* values 0.0015–<0.0001) or marbofloxacin (1.1–2.6 log_10_, 86.8–99.8% kill, *p* values 0.0111–0.0010). Statistically significant differences were seen for treatment (*p* = 0.0082), time (*p* < 0.0001) and treatment-by-time (*p* < 0.0001).

**Figure 4:**
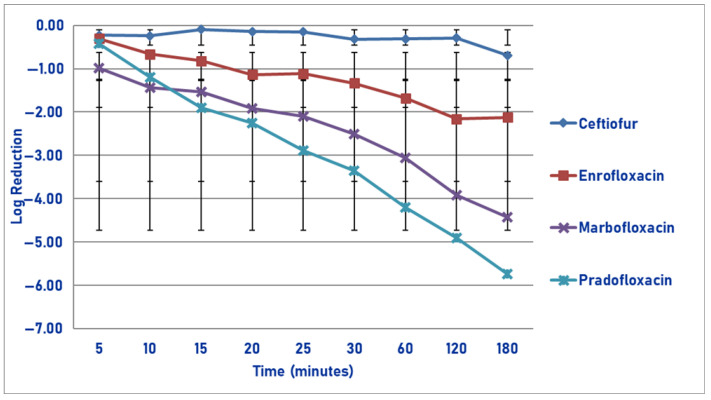
Log reduction in *Actinobacillus pleuropneumonia* (Isolates Averaged) by 4 antimicrobial agents—T_issuemax_.

Following exposure to the maximum tissue drug concentration, pradofloxacin killed 94.4% of cells following 15 min of drug exposure, compared to 12.2% for ceftiofur, 84.4% for enrofloxacin, and 94.9% for marbofloxacin (Figure 4). Pradofloxacin killed 99.9% of cells following 60 min of drug exposure and 100% of cells after 180 min of drug exposure. By comparison and after 60 and 180 min of drug exposure, ceftiofur killed 35.4 and 57.7% of cells; enrofloxacin killed 97.5 and 99.2% of cells; and marbofloxacin killed 99.9 and 99.99% of cells. Statistically significant differences in kill between the agents were not seen following 5, 10, and 15 min of drug exposure. Following 20–180 min of drug exposure, pradofloxacin (2.3–5.8 log_10_, 97.4–100% kill) killed more cells than ceftiofur did (0.1–0.7 log_10_, 20.6–57.7% kill, *p* values from 0.0135–<0.0001). Following 30–180 min of drug exposure, marbofloxacin (2.5–4.4 log_10_, 99.6–99.99% kill) killed more cells than ceftiofur did (0.3–0.7 log_10_, 4.9–57.7% kill, *p* values from 0.0150–<0.0001). Following 30–180 min of drug exposure, pradofloxacin killed more cells than enrofloxacin did (1.3–2.1 log_10_, 94.4–99.2% kill, *p* values 0.0415–<0.0001). Following 180 min of drug exposure, marbofloxacin killed more cells than enrofloxacin did (*p* = 0.0129). Statistically significant differences were seen for treatment (*p* = 0.0008), time (*p* < 0.0001), and treatment-by-time (*p* < 0.0001).

### 3.2. Pasteurella multocida

**Figure 5:**
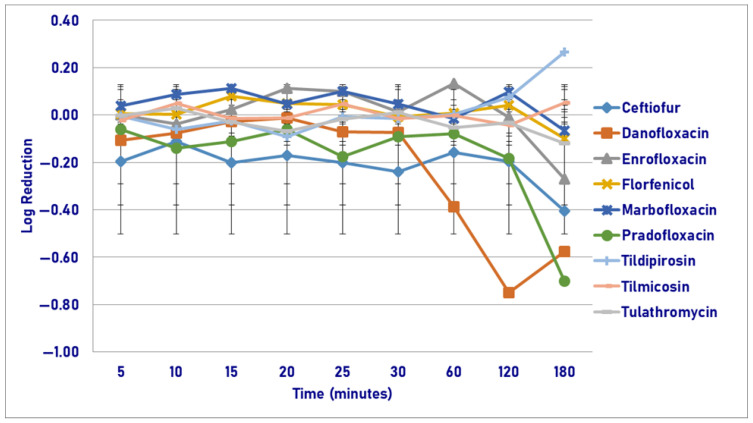
Log reduction in *Pasteurella multocida* (Isolates Averaged) by 9 antimicrobial agents—MIC.

Following exposure to the MIC drug concentration, pradofloxacin killed 62.7% of cells following 180 min of drug exposure as compared to 17.9–44.8% kill by ceftiofur, danofloxacin, enrofloxacin, and marbofloxacin, and growth in the presence of the other drugs (Figure 5). No statistically significant differences were seen between the agents following any time point up to and including 120 min of drug exposure. Following 180 min of drug exposure, pradofloxacin (0.7 log_10_, 62.7% kill) killed more cells than tildipirosin did (growth, *p* < 0.0001). A statistically significant difference was seen for time (*p* = 0.0089) and treatment-by-time (*p* = 0.0078).

**Figure 6:**
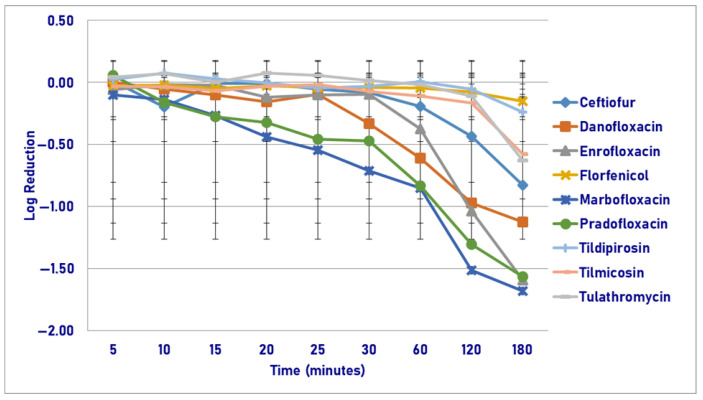
Log reduction in *Pasteurella multocida* (Isolates Averaged) by 9 antimicrobial agents—MPC.

Following exposure to the MPC drug concentration, pradofloxacin killed between 59.4 and 61.1% of cells following exposure to the MPC drug concentration and after 60–180 min of drug exposure (Figure 6). By comparison, ceftiofur, florfenicol, tildipirosin, tilmicosin and tulathromycin killed between 9 and 64.6% of cells following 120–180 min of drug exposure. Danofloxacin, enrofloxacin and marbofloxacin killed between 56.8 and 96.8% of cells following 60–180 min of drug exposure. Statistically significant differences in kill were not seen between the agents following 5–60 min of drug exposure. Following 120 min of drug exposure, marbofloxacin (1.5 log_10_, 81.9% kill) killed more cells than did florfenicol (0.1 log_10_, 22.7% kill, *p* = 0.0593) and tildipirosin (0.1 log_10_, 99% kill, *p* = 0.0397). Following 180 min of drug exposure, marbofloxacin (1.7 log_10_, 85.8% kill) killed more cells than florfenicol (0.2 log_10_, 27.7% kill, *p* = 0.0167) and tildipirosin did (0.2 log_10_, 34.8% kill, *p* = 0.0438). A statistically significant difference was seen for treatment (*p* = 0.0005), time (*p* < 0.0001), and treatment-by-time (*p* = 0.0007).

**Figure 7:**
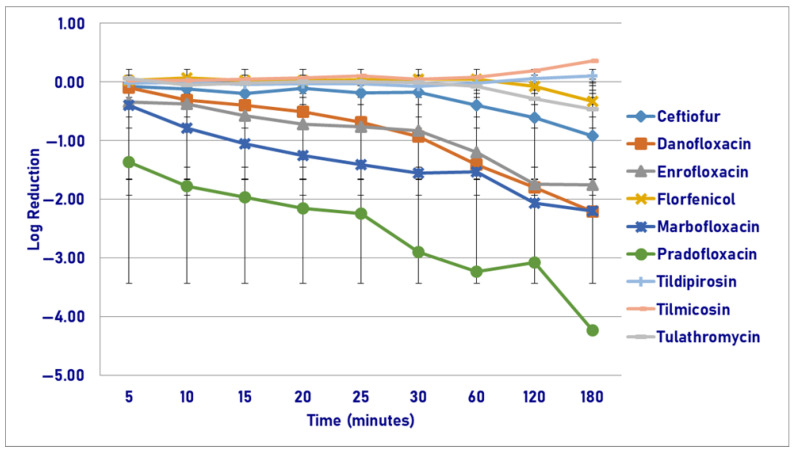
Log reduction in *Pasteurella multocida* (Isolates Averaged) by 9 antimicrobial agents—C_max_.

Following exposure to the maximum plasma drug concentration (C_max_), pradofloxacin killed 68.7–96.9% of cells following 5–30 min of drug exposure, 19.5–85.9% for danofloxacin, 51.5–81.4% for enrofloxacin, and 46.2–95.5% for marbofloxacin (Figure 7). The percentage of cells killed increased to 96.9–98.8% for all 4 quinolones following 180 min of drug exposure. Following 180 min of drug exposure, 24.6–87.3% of cells were killed by ceftiofur (87.3% kill), florfenicol, and tulathromycin. Growth occurred in the presence of tildipirosin and tilmicosin. Statistically significant differences in killing were not seen between agents following 5–15 min of drug exposure. Following 20 min of drug exposure, pradofloxacin (2.2 log_10_, 87.4% kill) killed more cells than florfenicol (growth, *p* = 0.0200), tildipirosin (0.03 log_10_, 5.4% kill, *p* = 0.0084), and tulathromycin did (growth, *p* = 0.0260). Following 25 min of drug exposure, pradofloxacin (2.3 log_10_, 77.1% kill) killed more cells than florfenicol (growth, *p* = 0.0078), tildipirosin (0.03 log_10_, 5.1% kill, *p* = 0.0030), tilmicosin (growth, *p* = 0.0249), and tulathromycin did (growth, *p* = 0.0105). After 30 min of drug exposure, pradofloxacin (2.9 log_10_, 96.9% kill) killed more cells than ceftiofur (0.2 log_10_, 14.1% kill, *p* < 0.0001), tilmicosin (growth, *p* < 0.0001), and tulathromycin did (0.01 log_10_, 1.6% kill, *p* < 0.0001). Following 60 and 120 min of drug exposure, pradofloxacin (3.1–3.2 log_10_, 96.9–97.7% kill) killed more cells than ceftiofur (0.4–0.6 log_10_, 53.9–72.7% kill, *p* < 0.0001, *p* = 0.0033), enrofloxacin (1.2–1.7 log_10_, 93.6–96.5% kill, *p* = 0.0439, *p* = 0.0271), florfenicol (growth-0.1 log_10_, *p* < 0.0001, *p* < 0.0001), tildipirosin (growth-0.02 log_10_, 4.7% kill, *p* < 0.0001 for both comparisons), tilmicosin (growth, *p* < 0.0001 for both comparisons) and tulathromycin did (0.1–0.03 log_10_, 13.3–43.9% kill, *p* < 0.0001 for both comparisons). Following 180 min of drug exposure, pradofloxacin (4.2 log_10_, 98.8% kill) killed more cells than ceftiofur (0.9 log_10_, 87.3% kill, *p* < 0.0001), enrofloxacin (1.8 log_10_, 96.9% kill, *p* = 0.0003), florfenicol (0.3 log_10_, 24.6% kill, *p* < 0.0001), marbofloxacin (2.2 log_10_, 97.8% kill, *p* = 0.0560), tildipirosin (growth, *p* < 0.0001), tilmicosin (growth, *p* < 0.0001) and tulathromycin did (0.5 log_10_, 48.9% kill, *p* < 0.0001). Danofloxacin (2.2 log_10_, 98.8% kill) killed more cells than tildipirosin (*p* = 0.0006) and tilmicosin did (*p* = 0.0003). Enrofloxacin killed more cells than tildipirosin (*p* = 0.013) and tilmicosin did (*p* = 0.0123). Marbofloxacin killed more cells than florfenicol (*p* = 0.0257), tildipirosin (*p* < 0.0001), and tilmicosin did (*p* < 0.0001). Statistically significant differences were seen for treatment, time, and treatment-by-time (*p* < 0.0001 for each variable).

**Figure 8:**
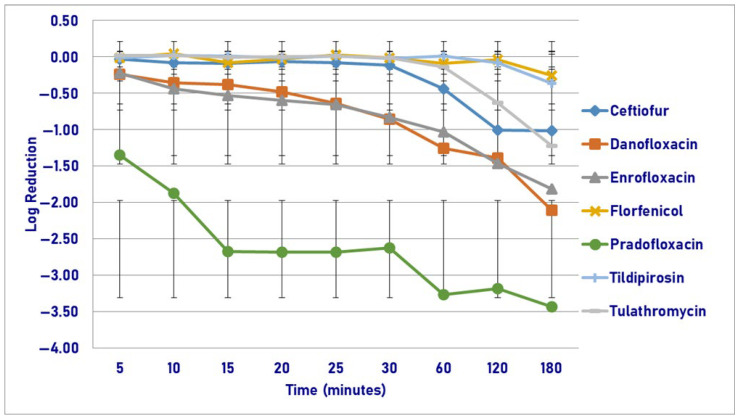
Log reduction in *Pasteurella multocida* (Isolates Averaged) by 7 antimicrobial agents—T_issuemax_.

Following exposure to the maximum tissue drug concentration (T_issuemax_), pradofloxacin killed 91.7% of cells following 5 min of drug exposure as compared to 24.4% for danofloxacin, 34.5% for enrofloxacin, and growth to 4.6% kill for the remaining agents (Figure 8). Killing in the presence of pradofloxacin increased to 99.1% following 20 min of drug exposure and to 99.3% following 180 min. By comparison, killing by danofloxacin and enrofloxacin increased to 58.5 and 97.7%, and 63.9% and 97.2%, following 20 and 180 min of drug exposure, respectively. For ceftiofur, this was 12.3% and 3.9%, florfenicol 5.6% and 34.8%, tildipirosin 3.2% and 33.7%, and tulathromycin, growth and 90.9%. No statistical differences in killing were seen between agents following 5 min of drug exposure. At all other time points (10, 15, 20, 30, 60, 120 and 180 min), pradofloxacin (1.9–3.4 log_10_, 97.3–99.3% kill) killed more cells than ceftiofur did (0.1–1.0 log_10_, 14.7–83.9% kill, *p* values 0.0001–<0.0001), danofloxacin (0.3–2.1% log_10_, 46.4–99.1% kill, *p* = 0.0480-*p* < 0.0001), enrofloxacin (0.2–1.8 log_10_, 53.7–97.2% kill, *p* values from 0.0024–<0.0001), florfenicol (0.01–0.3 log_10_, 2.0–34.8% kill, *p* values all at <0.0001 for each time point), tildipirosin (growth—0.4 log_10_, growth—33.7% kill, *p* values < 0.0001 for all comparisons) and tulathromycin (growth—1.2 log_10_, growth—90.9% kill, *p* values < 0.0001) for all comparisons). Following 120 and 180 min of drug exposure, enrofloxacin (1.5–1.8 log_10_, 91.7–97.2% kill) killed more cells than florfenicol did (0.04–0.3 log_10_, 6.8–34.8% kill, *p* = 0.0479 and *p* = 0.0103). Following 10 min of drug exposure, danofloxacin showed different results (1.4–2.1 log_10_, 97.7–99.1% kill). Enrofloxacin killed more cells than tildipirosin did following 180 min of drug exposure (*p* = 0.0012). Statistically significant differences were seen for time, treatment, and treatment-by-time (*p* < 0.0001 for each variable).

### 3.3. Streptococcus suis

**Figure 9:**
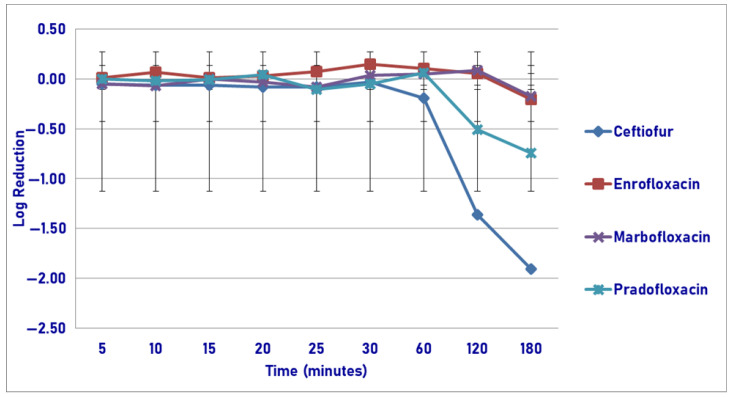
Log reduction in *Streptococcus suis* (Isolates Averaged) by 4 antimicrobial agents—MIC.

Following exposure to the MIC drug concentration, pradofloxacin killed 68.2–66.8% of cells following 120 and 180 min of drug exposure as compared to 94.3–98.7% for ceftiofur, growth to 18.2% for enrofloxacin, and growth to 2.8% for marbofloxacin (Figure 9). No statistical differences in killing were seen between agents following 5–60 min of drug exposure. Following 120 and 180 min of drug exposure, ceftiofur (1.4–1.9 log_10_, 94.3–98.7% kill) killed more cells than enrofloxacin (growth—0.2 log_10_, growth to 18.2% kill, *p* values < 0.0001 for both comparisons), marbofloxacin (growth—0.2 log_10_, growth—2.8% kill, *p* values < 0.0001 for both comparisons) and pradofloxacin did (0.5–0.7 log_10_, 68.2–66.8% kill, *p* values 0.0019 and <0.0001, respectively). Regarding time, statistically significant differences were seen in treatment (*p* = 0.0111), time (*p* < 0.0001), and treatment-by-time (*p* < 0.0001).

**Figure 10:**
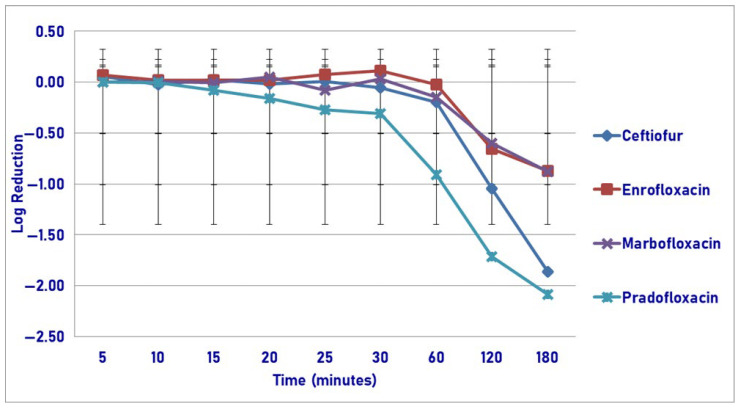
Log reduction in *Streptococcus suis* (Isolates Averaged) by 4 antimicrobial agents—MPC.

At the MPC drug concentration, pradofloxacin killed 87.7% of cells following 60 min of drug exposure, which increased to 98.0 and 99.2% kill following 120 and 180 min of drug exposure (Figure 10). By comparison, at those 3 time points, ceftiofur killed 30.9, 72.3, and 96.5% of cells, enrofloxacin killed 4.4, 72.3, and 75.4% of cells, and marbofloxacin killed 21.8, 68.3, and 88.3% of cells. Statistically significant differences in kill between the 4 drugs was not seen following 5–60 min of drug exposure. Following 120–180 min of drug exposure, ceftiofur (1.0–1.9 log_10_, 72.3–96.5% kill) killed more cells than enrofloxacin (0.7–0.9 log_10_, *p* values < 0.0001 for both comparisons) or marbofloxacin did (0.6–0.9 log_10_, 68.3–88.3% kill, *p* < 0.0001 for both comparisons). Pradofloxacin (1.7–2.1 log_10_, 98.0–99.2% kill) killed more cells than ceftiofur did following 120 (*p* = 0.0019) and 180 (*p* < 0.0001) min of drug exposure. Statistically significant differences were seen for time (*p* < 0.0001) and treatment-by-time (*p* < 0.0078).

**Figure 11:**
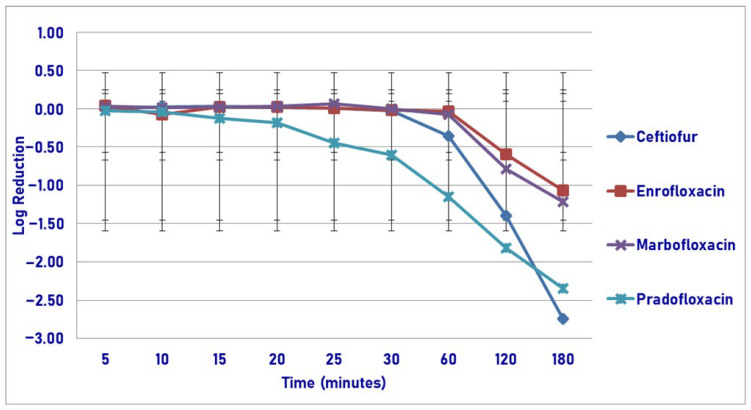
Log reduction in *Streptococcus suis* (Isolates Averaged) by 4 antimicrobial agents—C_max_.

At the maximum serum drug concentration, following 30, 60, 120 and 180 min of drug exposure, pradofloxacin killed 68.3, 92.4, 97.8 and 99.4% of cells as compared to ceftiofur (4.2, 43.9, 94.3 and 99.5% kill), enrofloxacin (growth, 7.0, 63.6 and 87.4% kill) and marbofloxacin (growth, 15.7, 80.0 and 92.6% kill) (Figure 11). Statistically significant differences in kill between the drugs were not seen following 5–30 min of drug exposure. Following 60 min of drug exposure, pradofloxacin (1.2 log_10_, 92.4% kill) killed more cells than enrofloxacin (0.04 log_10_, 7.0% kill, *p* = 0.0111) and marbofloxacin did (0.1 log_10_, 15.7% kill, *p* = 0.0429). After 120 min of drug exposure, pradofloxacin (1.8 log_10_, 97.8% kill) killed more cells than enrofloxacin did (0.6 log_10_, 63.6% kill, *p* = 0.0023). Following 180 min of drug exposure, ceftiofur (2.7 log_10_, 99.5% kill) killed more cells than enrofloxacin (1.1 log_10_, 87.4% kill, *p* < 0.0001) and marbofloxacin (1.2 log_10_, 92.6% kill, *p* < 0.0001). Pradofloxacin (2.4 log_10_, 99.4% kill) killed more cells than enrofloxacin (*p* = 0.0022) and marbofloxacin did (*p* = 0.0455). Statistically significant differences were seen in treatment (*p* = 0.0079), time (*p* < 0.0001), and treatment-by-time (*p* < 0.0001).

**Figure 12:**
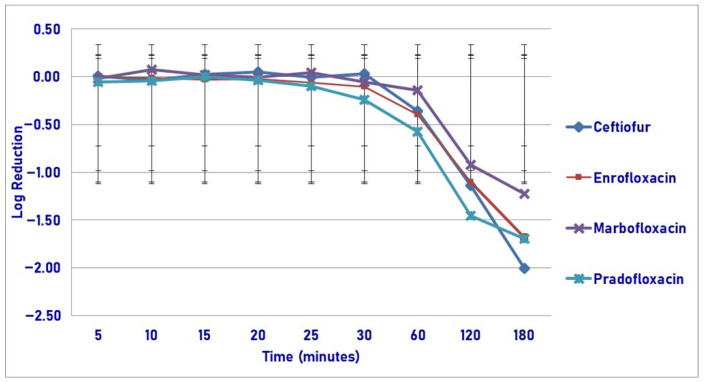
Log reduction in *Streptococcus suis* (Isolates Averaged) by 4 antimicrobial agents—T_issuemax_.

At the maximum tissue drug concentration and following 60, 120 and 180 min of drug exposure, pradofloxacin killed 71.6, 93.2 and 97.1% of cells, compared to ceftiofur (46.2, 79.8 and 98.1% kill), enrofloxacin (58.9, 91.2 and 97.2% kill) and marbofloxacin (18.5, 87.7 and 95.4% kill) for the same three time points (Figure 12). Statistically significant differences in kill between the drugs were not seen at any time point measured. A statistically significant difference was seen for time (*p* < 0.0001).

## 4. Discussion

In porcine respiratory disease complex, primary viral pathogens include porcine reproductive and respiratory syndrome virus, swine influenza virus, and porcine circovirus type 2, whereas respiratory coronavirus is considered a secondary viral pathogen. Of bacteria, *A. pleuropneumoniae* and some strains of *P. multocida* are considered primary pathogens, while most strains of *P. multocida* are considered secondary pathogens. *P. multocida* specifically has a number of identified virulence factors (i.e., capsule, lipopolysaccharide, surface adhesions, etc.) that facilitate pathogenesis [17]. Bacterial pathogens (e.g., *A. pleuropneumoniae*, *P. multocida*, *S. suis*, *G. parasuis*) cause the appearance of fibrin on the pleural surface of the lung, giving rise to fibrous pleurisy with chronic disease [18]. Antimicrobials for bacterial infection remain the cornerstone for therapy in infected animals, and Brockmeier and colleagues stated that judicious and timely use of appropriate antibiotics (and vaccination protocols) is typically used to control respiratory disease [19]. Dee and colleagues, in a unique approach to studying antibiotic-treated versus antibiotic-free regimens, showed the negative impact of antibiotic-free treatments in pigs [20]. Three groups of pigs were investigated: Group 1 (n = 702) received mass antibiotic medications on days 4 and 21 and judicious antibiotic therapy thereafter in water or feed by individual injection, Group 2 (n = 675) was similar to group 1 with mass treatment on day 4 and no subsequent therapeutic feed medication, and Group 3 (n = 702) was antibiotic-free. All animals received a modified−live PRRSV vaccine 3 days after weaning. Pigs were contact challenged with the virulent PRRSV lineage 1 strain (174) four weeks after vaccination. Antibiotics were argued to be important for bacterial co-infections. Statistically significant differences were not seen between groups 1 and 2 but they were in group 3 when considering average daily gains and mean feed conversion ratio at finishing, and favoured the antibiotic treatment groups. Mortality and removal were significantly lower in groups 1 (20.94%) and 2 (24.89%) when compared to the antibiotic-free group (57.98%). The authors argued that antibiotic-free production strategies may leave pigs at risk of severe clinical disease, and judicious antibiotic use can significantly improve animal health. The authors also commented that efficacious disease prevention strategies may reduce the need for antibiotics, thereby maximizing efficacy and reducing the risk for antimicrobial resistance over time.

Limited published in vitro susceptibility data exist for *S. suis* and pradofloxacin. Risser et al. reported on 254 *S. suis* from the lung with an MIC_50_ and MIC_90_ of 0.06 µg/mL and 0.25 µg/mL, respectively (MIC range was 0.015–8 µg/mL); for 30 isolates from the brain, the values were 0.06 g/mL, 0.12 µg/mL, and 0.015–0.12 µg/mL, respectively. The MIC values of the strains used in this study ranged from 0.031 to 0.125 µg/mL and were consistent with those reported by Risser et al. [21].

In this study, we report on the killing of swine bacterial pathogens by pradofloxacin and comparator antimicrobial agents in a 3 h kill assay, which provides a valuable insight into the in vitro killing of bacteria following the first 3 h of drug exposure. Pradofloxacin was bactericidal against the bacterial pathogens tested, resulting in 87.7–99.2%, 92.4–99.4%, and 71.6–97.1% killing of *S. suis* strains following 60–180 min exposure to the MPC, maximum serum, and maximum tissue drug concentration, respectively. The values for *A. pleuropneumoniae* were 72–99.9%, 99.99–100%, and 99.9–100%, and for *P. multocida* they were 59.4–61.1%, 96.9–99.0%, and 99.8–99.3%, respectively. For enrofloxacin and marbofloxacin against *S. suis*, 75.4–97.2% and 88.3–95.4% of cells were killed following 180 min of drug exposure to the 3 drug concentrations mentioned above. Similarly, for ceftiofur, 72.3–99.5% of cells were killed following 120–180 min of drug exposure. Ceftiofur killed fewer *A. pleuropneumoniae* cells (54.8–78.8%) following 180 min of drug (MPC, maximum serum, and maximum tissue drug concentration) than enrofloxacin (99.2–99.9%) or marbofloxacin did (91.4–99.99%). Finally, for *P. multocida,* ceftiofur, enrofloxacin, and marbofloxacin killed 37.9–87.3%, 96.9–97.2%, and 85.8–97.8% following 180 min of drug exposure to the three aforementioned drug concentrations (MPC and maximum plasma only for marbofloxacin). For the remaining agents (florfenicol, tildipirosin, tilmicosin, and tulathromycin) and following 180 min of drug exposure, no agent exceeded 90.9% kill (growth to 90.9% kill) at any of the aforementioned drug concentrations.

In a previous study from our laboratory, we reported similar observations to those reported here for *P. multocida* strains from cattle tested against the same antimicrobials, bacterial density, and timelines (i.e., over 180 min) [11]. In another study, higher bacterial densities of *P. multocida* strains than reported in this study, and with kill measured over 24 h, showed similar trends in killing for the same antimicrobial as reported here [22]. These various in vitro kill studies show pradofloxacin to be rapidly bactericidal, and bactericidal activity was seen for the other quinolones tested against ceftiofur. Bacteriostatic activity was typically seen for florfenicol as well as the macrolide and macrolide-like agents.

Pradofloxacin is the most recently approved antimicrobial agent for treating swine respiratory disease. The eradication of bacteria that cause infection is necessary for clinical cure [23]. Pradofloxacin has a number of characteristics that are favourable. First, against the target pathogens, MIC values are low (≤0.016 µg/mL); second, MPC values are at or below the susceptibility breakpoint for *A. pleuropneumoniae* and *P. multocida* [16,24]; third, it is a dual-targeting compound with simultaneous lethal inhibition of DNA gyrase (topoisomerase II) and topoisomerase IV, which suggests a low likelihood for resistance selection; and fourth, dual targeting is reported to reduce the likelihood of resistance selection from susceptible bacterial populations [25]. Spontaneous first-step resistant mutant subpopulations are known to occur between bacterial densities ranging from 10^7^ to 10^9^ cfu/mL [26]—a density of cells reported to occur during infection. A single target antibiotic could select for bacterial subpopulations with reduced susceptibility to the drug. For a dual-targeting drug, two simultaneous mutations would be required for resistance—a rare event requiring an organism density of 10^−14^ or greater (1 × 10^−7^ × 1 × 10^−7^ = 10^−14^). In this study, we did not investigate or prove dual-targeting activity; however, other investigations describe the high affinity of pradofloxacin for both topoisomerase IV and DNA gyrase [27,28,29,30]. Additionally, higher densities of bacteria than those used here would be required to test inhibition or killing of the first-step mutant by pradofloxacin or other quinolones. Regardless, judicious antimicrobial use is a necessary component for preventing antimicrobial resistance. For the strains tested and under the experimental conditions used, pradofloxacin was rapidly bactericidal. It has a spectrum of activity suitable for treating swine with bacterial infection. Dual targeting activity may reduce the role of resistance selection

This study has some limitations worthy of consideration. First, this is an in vitro study, and the correlation between an in vitro observation and clinical outcome may not be absolute. Stratton indicated that the incorporation of PK/PD indices has greatly improved the correlation between in vitro susceptibility and in vivo clinical effectiveness [31]. Doern and Brecher reported that the clinical predictive value of a susceptibility result is excellent at >90%; however, the prediction of failure was substantially less at <35% [32]. Second, protein binding was not measured in our experiments and has been reported to impact antimicrobial activity [33]. Third, kill was measured for 180 min, and longer exposure to some antimicrobial agents may have affected the log_10_ (% kill) reduction in viable cells. Additionally, 3 h may not be long enough to detect organism regrowth should it occur. Firsov et al. showed that, depending on the drug concentration used, regrowth occurred within 3 h (lower drug concentration) and upwards of 10–20 h (higher drug concentration) in experiments using ciprofloxacin and *E. coli* [34]. Fourth, the use of different strains with different MIC or MPC values may yield different results for some drugs. Fifth, we used three strains of each organism, but a larger number of strains may be necessary to fully capture wild-type strain variability. In previous reports [10,11,22], three strains of each organism have been acceptable with this limitation.

## 5. Conclusions

Against swine respiratory disease bacterial pathogens (*A. pleuropneumoniae*, *P. multocida*, *S. suis*), pradofloxacin was rapidly bactericidal at clinically relevant drug concentrations, resulting in 99–100% kill at the maximum plasma and maximum tissue drug concentrations. Pradofloxacin appears to be a useful new agent for treating swine respiratory disease caused by bacteria.

## Figures and Tables

**Table 1:** Comparative MIC, MPC, and therapeutic drug concentration values for antimicrobial agents.

	Isolates						C_max_ (µg/mL)	T_issuemax_ (µg/mL)
	#3		#15		#22			
	MIC	MPC	MIC	MPC	MIC	MPC		
*Actinobacillus pleuropneumoniae*								
Ceftiofur	0.031	0.063	0.016	0.063	0.063	0.125	16	2.64
Enrofloxacin	0.063	0.5	0.031	0.25	0.031	0.5	1.4	4.6
Marbofloxacin	0.031	0.063	0.031	0.063	0.031	0.25	1.62	1.74
Pradofloxacin	0.016	0.125	0.008	0.031	0.008	0.125	4/2.2	0.81
***P. multocida***	**#5**		**#6**		**#14**			
Ceftiofur	0.002	0.125	0.002	0.125	0.002	0.25	6.9	2.64
Danofloxacin	0.016	0.063	0.016	0.063	≤0.008	0.063	0.4	1.68
Enrofloxacin	0.008	0.063	0.004	0.063	0.008	0.031	1.9	4.6
Florfenicol	0.5	1	0.25	1	0.5	0.5	4.5	2.94
Marbofloxacin	0.016	0.125	0.008	0.125	0.016	0.25	1.5	NT
Pradofloxacin	≤0.008	0.031	0.031	0.031	0.004	0.25	2.64	0.81
Tildipirosin	1	4	0.5	4	0.5	4	0.767	14.77
Tilmicosin	4	32	2	8	2	4	0.25	NT
Tulathromycin	0.5	2	0.25	1	0.5	1	0.6	3.2
***S. suis***	**#1**		**#2**		**#3**			
Ceftiofur	0.063	0.5	0.25	0.5	0.063	0.5	18.2	6.03/12.09
Enrofloxacin	0.5	2	0.125	1	0.5	2	1.4	4.6
Marbofloxacin	1	2	0.25	1	0.5	2	1.62	1.74
Pradofloxacin	0.125	2	0.031	0.5	0.063	2	2.64	0.8

MIC = minimum inhibitory concentration; MPC = mutant prevention concentration; µg/mL = microgram per millilitre; NT = not tested.

## Data Availability

The data that support the findings of this study are available from the corresponding author upon reasonable request.
